# Footwear habits and general health in middle-aged women: a retrospective ten year study

**DOI:** 10.1186/1757-1146-7-S2-A2

**Published:** 2014-11-14

**Authors:** Catherine Bowen, David Culliford, Nigel Arden

**Affiliations:** 1 Faculty of Health Sciences, University of Southampton, Southampton, UK; 2 Nuffield Department of Orthopaedics, Rheumatology and Musculoskeletal Sciences, University of Oxford, Oxford, UK; 3 Faculty of Medicine, University of Southampton, Southampton, UK

## Background

Success of footwear as a modifiable intervention is complex and various qualitative studies have revealed that for women in particular shoes form an important part of clothing, self-identity and self-esteem. Whilst it is accepted that heel height may be an important differentiating characteristic of shoes that affect biomechanical function there is very little epidemiological longitudinal data that investigates other factors such as health and well-being. The objective of this study was to describe the footwear habits and relationship of this to general health in middle-aged women over a 10-year period.

## Methods

A retrospective, longitudinal, cohort design was used to investigate footwear type, heel height, duration of daytime and evening hours shoe wear and general health. Data was prospectively collated over 20 years for women from the general population (The 1000 Women Study). From a baseline of 1003 female participants, 811 were reviewed at year 10 (Y10) & 15, and 533 year 20 (Y20). Median age at Y10 was 61 years (57-67).

## Results

21.6% women reported foot pain at Y10 that increased to 26.6% 5 years later (Y15). At year 10, 84% (n=682) of women were wearing heels higher than two inches, and by year 20 this dropped to 53% (n=292). The most common type of high heels at Y10 and 20 were stilettos, then court shoes. The majority of women reported 31-40 daytime hours and 1-10 evening hours of wearing high heels at Y10 but less at Y20 (1-10 hours daytime and evening). Chi-squared analysis showed that wearing high heels was significantly associated with general good health (p=0.002).

## Conclusion

Middle aged women often present with foot pain, yet regularly wear high heels for long periods during the day and evening, although this reduces with age. It appears that the wearing of high heels may be associated with general good health. However further analysis and adjustment of data for confounding factors is required to determine the real effect of high heels on general health.

